# Broccoli Myrosinase cDNA Expression in *Escherichia coli* and *Saccharomyces cerevisiae*

**DOI:** 10.3390/biom12020233

**Published:** 2022-01-30

**Authors:** Carolina Curiqueo, Andrea Mahn, Antonio Castillo

**Affiliations:** 1 Departamento de Biología, Facultad de Química y Biología, Universidad de Santiago de Chile (USACH), Av. L. B. O’Higgins 3363, Estación Central, Santiago 9170022, Chile; carolina.curiqueo@usach.cl; 2 Departamento de Ingeniería Química, Facultad de Ingeniería, Universidad de Santiago de Chile (USACH), Av. L. B. O’Higgins 3363, Estación Central, Santiago 9170022, Chile; andrea.mahn@usach.cl

**Keywords:** broccoli myrosinase, cDNA expression, *Escherichia coli*, *Saccharomyces cerevisiae*

## Abstract

Myrosinases (EC 3.2.1.147) are enzymes known for the generation of hydrolysis products that have a potential beneficial effect on human health. Their reaction mechanisms are widely studied, in order to improve and optimize secondary metabolite production processes. In this work, kinetic and biochemical properties of the broccoli myrosinase enzyme produced from its cDNA cloned in *Escherichia coli* and *Saccharomyces cerevisiae* were investigated. The results revealed that the thermal stability of the enzyme produced in *S. cerevisiae* was slightly higher (30 to 60 °C) than that of myrosinase produced in *E. coli* (20 to 50 °C). The effect of pH on the enzymatic activity was similar in both enzymes, with pH 3 being the optimum value under the reaction conditions used. The kinetic behavior of both enzymes was adjusted to the Michaelis–Menten model. The catalytic efficiency was up to 4 times higher in myrosinase produced in *S. cerevisiae*, compared to myrosinase produced in *E. coli*. The glycosylations present in the enzyme would be related to the formation of a dimeric quaternary structure and would not play an essential role in enzymatic activity, since both enzymes were biologically active. These results will probably allow the development of strategies for the production of bioactive metabolites of medical interest.

## 1. Introduction

Myrosinases (β-thioglucosidase glucohydrolase, EC 3.2.1.147) are glycoproteins found mainly in the *Brassicaceae* family and catalyze the hydrolysis of various secondary metabolites called glucosinolates (GSL) [1]. In the *Brassicaceae* family, more than 130 types of GSL have been described, from which a wide variety of myrosinase hydrolysis products with different chemical and biological properties can be generated, including those that have a potential beneficial effect on human health [2,3]. For this reason, the mechanisms of action of myrosinases are widely studied, in order to improve and optimize the production processes of these secondary metabolites. Catalysis occurs by cleavage of the β-thioglucosidic bond and the equimolar release of a β-D-glucose molecule, forming an unstable intermediate called thiohydroxamate-O-sulfonate, which through the release of a sulfate anion generates isothiocyanates, thiocyanates, epithionitriles or nitriles [4]. The formation of these hydrolysis products not only depends on the type of hydrolyzed GSL, but also depends on the pH, the presence of ferrous cations, epithiospecifier proteins (ESP), nitrile-specifier proteins (NSPs) and thiocyanate-forming proteins (TFPs) [5,6,7,8]. Among the various hydrolysis products, we can find isothiocyanates, which are of commercial interest because they have multiple beneficial properties for human health [9,10,11].

Several research groups have studied the biochemical and kinetic characteristics of myrosinases obtained from different sources [12,13,14,15,16,17,18,19]. Structural studies using X-ray diffraction have made it possible to elucidate the crystal structure of myrosinase from *Sinapis alba*, where it was revealed that the monomeric subunit folds to form a highly glycosylated dimer stabilized by Zn^2+^, with a catalytic site in each monomer of the protein, and in turn each monomer folds with an α and β barrel-like structure. In addition, this active site contains two pockets, one of them is adapted to accept the negatively charged group (sulfonated oxime), due to the presence arginine and lysine residues, and the other pocket is specific for the β-D-glucose portion of the substrate [20]. The kinetic behavior of most myrosinases adjusts to the Michaelis–Menten model. Enzyme activity increases at low ascorbic acid concentrations and in the presence of divalent cations such as Mg^2+^. Finally, myrosinases are stable in a pH range of 4 to 7 and at temperatures between 30 and 60 °C [7,21,22].

Despite the various studies that exist on myrosinase of different origins, in 2014 a group of researchers became interested in the study of the biochemical and kinetic characteristics of broccoli myrosinase, because this vegetable contains a considerable amount of glucoraphanin and the hydrolysis of this substrate generates a bioactive metabolite called sulforaphane, which has outstanding prophylactic or healing properties for important human pathologies [23]. The enzyme was described with a homotrimer quaternary structure, with a molecular mass of approximately 157 kDa and with a kinetic behavior that adjusts to the substrate inhibition model [24]. Subsequently, in 2018, the myrosinase cDNA was cloned from the total RNAs obtained from broccoli inflorescences, obtaining a sequence of 1749 nucleotides (nt) with an open reading frame (ORF) of 1647 nt. With these data, a three-dimensional structural model of the monomeric subunit of the enzyme was built, which was used to perform molecular docking simulations with the substrate’s glucoraphanin and sinigrin [25]. The data of the kinetic parameters obtained were adjusted to a model of two binding sites of the substrate, one of them being the active site and the other a regulatory site [25], which confirmed the inhibition model per substrate proposed previously. The broccoli enzyme is the only myrosinase for which a kinetic behavior has been described that adjusts to the substrate inhibition model. Therefore, it would be interesting to study the role that plays post-translational modifications, particularly glycosylations, on their enzymatic activity, formation of quaternary structure, or thermal stability. In addition, obtaining high amounts of the myrosinase enzyme, with high specific activity, could greatly facilitate further kinetic and structural studies. In this context, the use of recombinant DNA techniques allows the overexpression of genes and the obtaining of large amounts of proteins. For this, there is a wide range of expression systems in different hosts, being prokaryotic systems useful in terms of production yields, low cost of the expression system, and easy manipulation of the microorganism for the production of eukaryotic proteins, despite the absence of post-translational modifications [26,27]. Additionally, the use of a eukaryotic system will allow to compare both gene products and determine the importance of post-translational modifications in the structure, stability and biological activity of the protein. In specific applications such as in the food industry, the use of prokaryotic or eukaryotic organisms generally recognized as safe (GRAS) is mandatory.

In this work, broccoli myrosinase was produced in *Escherichia coli* transformed with a pET expression vector, to which the myrosinase cDNA was inserted in front of the promoter of the T7 phage RNA polymerase. Induction with IPTG generates high levels of transcripts and consequently high levels of the gene product [28]. Broccoli myrosinase was also produced in *Saccharomyces cerevisiae* through the use of a self-selection system and the sequential induction of the GAL1 promoter present in the pMG1 vector [29]. The use of prokaryotic and eukaryotic hosts in the expression of myrosinase genes from other origins has been successful and has contributed to obtaining structural information, in enzyme immobilization studies and in genomic studies [30,31,32].

The main purpose of this work is to describe for the first time a biochemical and kinetic characterization of broccoli myrosinase produced in *E. coli* and *S. cerevisiae*, in order to compare these results with those previously obtained with the native enzyme purified directly from broccoli. The formation of quaternary structure, thermal stability and kinetic behavior of the enzyme was analyzed. In addition, the role of glycosylations of the enzyme produced in *S. cerevisiae* through deglycosylation and how it affects the properties mentioned above was investigated.

The results presented in this paper may be used to overproduce recombinant broccoli myrosinase in a GRAS (generally regarded as safe) organism such as *S. cerevisiae* and thus be able to design new strategies to obtain beneficial products for human health, such as sulforaphane, which has tremendous pharmacological potential for the prophylaxis or treatment of some severe human pathologies.

## 2. Materials and Methods

### 2.1. Obtaining Recombinant Clones

The directional cloning strategy was used to subclone the myrosinase cDNA into the bacterial vector pET-22b(+) (Novagen) and into the yeast vector pMG1, which was kindly donated by Dr. Marco Geymonat from Division of Stem Cell Biology and Developmental Genetics, National Institute for Medical Research, Mill Hill, London, UK. The myrosinase cDNA was obtained from a pJET vector (Thermo Fisher Scientific, Waltham, MA, USA) by digestion with the enzymes *Bam*HI and *Xho*I. *E. coli* BL21(DE3) and *S. cerevisiae* MGY70 were transformed with the recombinant vector by the calcium chloride method [33] and the lithium acetate method [34], respectively. The confirmation of the recombinant clones was carried out by PCR using the forward primer: 5′-TGCACCAGGTCGATGTTCTC-3′ and the reverse primer: 5′-AAGGGTCGCCGTCTTTGGTT-3′ to amplify the myrosinase cDNA. The recombinant clones were named *E. coli* BL21(DE3)-myr and *S. cerevisiae* MGY70-myr.

### 2.2. Expression of the cDNA Encoding Myrosinase in E. coli and S. cerevisiae

From a preinoculum of *E. coli* BL21(DE3)-myr, a 1:100 dilution was made in LB medium (1% tryptone, 1% yeast extract, 1% sodium chloride) containing 100 μg/mL ampicillin, up to an OD 0.5–0.6 (~4 h) at 600 nm. After this time, 1 mM IPTG was added to the culture medium and 3 h later the cells were centrifuged at 4500× *g* for 5 min at 4 °C. The expression of the cDNA in *S. cerevisiae* MGY70-myr was performed according to the previously described protocol [35], with the only modification that 1% galactose induction was carried out for 24 h.

### 2.3. Purification of Myrosinase Enzyme

The cell pellet previously obtained from the induced culture of *E. coli* BL21(DE3)-myr, was resuspended in 300 μL of Tris·HCl buffer pH 7 containing 10 μg/mL of lysozyme and incubated for 15 min at room temperature. It was then sonicated for 2 min with 4 pulses of 20 s at 40% power and centrifuged at 7000× *g* for 15 min. The supernatant was filtered through a filter with a pore size of 0.22 µm in diameter, and was subsequently loaded on an NTA-Ni column (Novagen), which was previously equilibrated with 15 mL of binding buffer (20 mM Imidazole, 20 mM sodium phosphate, 500 mM NaCl, pH 7.4). Then 1 mL of filtered protein extract (4.3 mg/mL) was loaded, and the eluate was collected. Subsequently, 5 mL of washing buffer (80 mM Imidazole, 20 mM sodium phosphate, 500 mM NaCl, pH 7.4) were loaded, and the recombinant protein was eluted with 5 mL of elution buffer (1 M Imidazole, 20 mM sodium phosphate, 500 mM NaCl, pH 7.4).

The cell pellet previously obtained from the induced culture of *S. cerevisiae* MGY70-myr, was resuspended in 300 μL of Tris HCl buffer pH 7, and sonicated for 2 min with 4 pulses of 20 s at 40% power, and then centrifuged at 7000× *g* for 15 min. The supernatant was filtered through a filter with a pore size of 0.22 µm in diameter and subsequently loaded onto an EZCatchTM GST-Spin column (BioVision, Milpitas, CA, USA). Purification was carried out following the manufacturer’s instructions.

The percentage yield of the purification process was defined in relation to the total enzymatic activity (U) in each purification stage, considering that the amount of total enzymatic activity in the crude extract is 100%. The quantification of total proteins and fractions eluted from the NTA-Ni column and EZCatchTM GST-Spin was performed by the method described [36].

### 2.4. SDS-PAGE and Western Blot

The protein fractions were resolved on 10% polyacrylamide gels under denaturing conditions (SDS-PAGE). Protein visualization was performed by Coomassie blue R-250 and silver nitrate staining. The molecular mass of the monomeric subunit of myrosinase was determined as a function of its migration distance, using the Precision Plus Protein Dual Xtra Standards (Bio-Rad, Hercules, CA, USA) protein markers.

For Western Blot analysis, the proteins resolved by SDS-PAGE were transferred to a nitrocellulose membrane (pore size 0.25 µm), using an electro blot chamber (Mini Trans-Blot^®^ Module, Bio-Rad, Hercules CA, USA). The membrane was incubated in a blocking solution containing 5% skim milk in TBS-T buffer (20 mM Tris·HCl pH 7, 0.1 M NaCl, 0.05% Tween-20), for 1 h at room temperature. The membrane was incubated for 2 h at room temperature and under constant shaking with the primary anti-His antibody conjugated to the HRP enzyme (1:1000) to detect the recombinant protein produced in *E. coli* BL21(DE3)-myr (Santa Cruz Biotechnology, Inc., Dallas, TX, USA) and the primary anti-GST antibody conjugated to the enzyme HRP (1:1000) to detect the recombinant protein produced in *S. cerevisiae* MGY70-myr. The development was carried out using autoradiography films and the WESTAR substrate (Cyanagen, Bologna, Italy).

### 2.5. Determination of the Molecular Mass of Native Myrosinase

Chromatographic runs were performed in a BioLogic LP System equipped with a fraction collector and the LP Data View software (Bio-Rad, Hercules, CA, USA). A 0.7 × 30 cm column (Bio-Rad, Hercules, CA, USA) was used, which was packed with 15 mL of Sephacryl S-200 HR (Sigma Aldrich, Schnelldorf, Germany). The calibration curve was constructed using the protein gel filtration kit (13.5–75 kDa) (Sigma Aldrich, Schnelldorf, Germany). Chromatography was performed loading 1 mL of the previously purified myrosinase fraction (1 mg/mL), and it was eluted at a flow of 0.3 mL/min using 0.2 M Tris·HCl buffer, pH 7.4.

### 2.6. Enzymatic Activity of Recombinant Myrosinase

The enzymatic activity of myrosinase was determined according to the protocol described by Li & Kushad, (2005) [13]. The reaction mixture contained 100 μL of protein (0.05 mg) in 800 μL of 33 mM sodium phosphate buffer, pH 7. This mixture was incubated at 30 °C for 3 min, then 100 μL of 0.1 mM sinigrin (Sigma-Aldrich, Schnelldorf, Germany) were added, and the myrosinase activity was determined by the rate of hydrolysis of sinigrin at an absorbance of 227 nm, for 1 min. The blank used contained the same mixture, but instead of using 100 µL of sinigrin, 100 µL of 33 mM sodium phosphate buffer, pH 7 was added. For the calculations, the equation described by Li & Kushad, (2005) [13] was used, with a molar extinction coefficient (ε) for sinigrin at 227 nm of 6784 M^−^^1^ cm^−^^1^, the path of light through of the cuvette (l) equal to 1 cm, the total volume of the reaction mixture (Vr) was 1 mL, and the volume of solution with enzyme (Ve) was equal to 0.1 mL. One unit of enzyme activity (U) is defined as the amount of enzyme that catalyzes the hydrolysis of 1 μmol of sinigrin per minute. All the tests were carried out in triplicate and the specific activity was expressed in units per milligram of protein (U/mg).

The kinetic constants were determined using the Lineweaver-Burk plot, plotting the double reciprocals of the initial rates versus substrate concentration. The point of intersection of the straight line with the ordinate axis corresponded to 1/V_max_, while the point of intersection with the abscissa axis corresponded to −1/K_m_ (Supplementary Material, Figure S1).

### 2.7. Effect of Temperature and pH on Myrosinase Activity

The broccoli myrosinase activity was determined at different conditions of temperatures (20 to 70 °C) and pH (2 to 8). The effect of pH was evaluated at 30 °C using glycine-HCl buffer (pH 2 and 3), acetate buffer (pH 4 and 5), sodium phosphate buffer (pH 6 and 7) and Tris·HCl buffer (pH 8). All measurements were carried out at 30, 60 and 70 °C, and 0.05 mg of proteins and 0.1 mM of sinigrin were used in each reaction.

### 2.8. Kinetic Characterization of Recombinant Myrosinase

The kinetic behavior of broccoli myrosinase produced in *E. coli* BL21(DE3)-myr and *S. cerevisiae* MGY70-myr was determined using the previously described protocol [13] at 30 °C, and in 33 mM sodium phosphate buffer pH 7, using different concentrations of sinigrin, which were 1, 5, 10, 25, 50, 100, 150, 200, 250, 300, and 350 μM. Experimental data were fitted to the Michaelis–Menten model and all measurements were performed in triplicate using 0.05 mg of proteins.

### 2.9. Deglycosylation and Proteolytic Digestion of Recombinant Myrosinase

Three treatments were carried out with myrosinase produced in *S. cerevisiae* MGY70-myr. Thrombin protease 500 U (GE Healthcare, Amersham, UK) was used for proteolytic digestion following the manufacturer’s recommendations. For each treatment, 0.1 mg of purified enzyme and 1 U of thrombin protease were used, the incubation was carried out for ~20 h. The deglycosylation of myrosinase was performed with Endoglycosidase H 10.000 U (Promega, Madison, WI, USA) following the manufacturer’s recommendations. Each treatment was carried out with 0.1 mg of purified enzyme, the reaction mixture was incubated for ~18 h at 37 °C. The protein preparation was resuspended in TE buffer (0.1 M Tris·HCl pH 7.5, 0.01 M EDTA), subsequently transferred to an Amikon filter (Merk Millipore, Darmstadt, Germany), and centrifuged at 4000× *g* for 5 min.

Three types of treatments were performed called: deglycosylated myrosinase with GST-tag, deglycosylated myrosinase without GST-tag, and glycosylated myrosinase without GST-tag. Subsequently, a size exclusion chromatography was performed in the same conditions described.

### 2.10. Statistical Analysis

Statistical analysis of the data was performed using GraphPad Prism 8.0.2 (GraphPad Software, San Diego, CA, USA). For the estimates of the constants K_m_, *k*_cat_ and V_max_, the standard error (SE) is reported, associated with each non-linear regression kinetic parameter using Michaelis–Menten. In addition, to confirm these data, a linear regression of the reciprocals of each sample was performed using Lineweaver-Burk. In the graphs, the error bars represent the distribution of the means of each sample, with a size of three replicates.

## 3. Results

### 3.1. Myrosinase cDNA Expression and Recombinant Protein Purification

**Figure 1:**
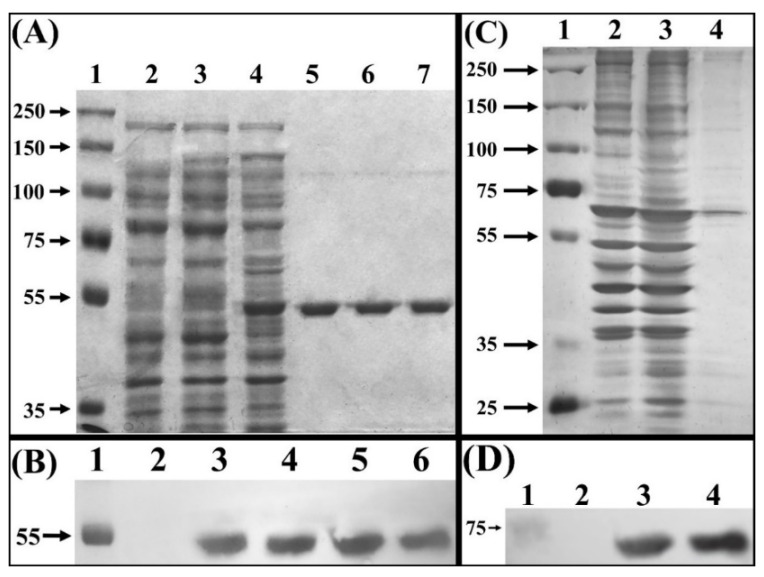
Electrophoretic separation of crude extract proteins. (**A**) SDS-PAGE of total proteins extract from *E. coli* BL21(DE3) stained with Coomassie blue. Lane 1, molecular mass marker (Precision Plus Protein Dual Xtra Standards). Lane 2, crude protein extract from strain *E. coli* BL21(DE3). Lane 3 crude protein extract from non-induced strain BL21(DE3)-myr. Lane 4, crude protein extract from *E. coli* BLD21(DE3)-myr induced with IPTG. Lanes 5, 6 and 7, fractions eluted from NTA-Ni column. (**B**) Western blot, using anti–His tag antibody. Lane 1, molecular mass marker (Precision Plus Protein Dual Xtra Standards). Lane 2, total proteins extract from BL21(DE3)-myr non-induced. Lane 3, total proteins extract from BL21(DE3)-myr induced with IPTG. Lanes 4, 5 and 6, fractions eluted from NTA-Ni column. (**C**) SDS-PAGE of total proteins from *S. cerevisiae* MGY70 stained with silver nitrate. Lane 1, molecular mass marker (Precision Plus Protein Dual Xtra Standards). Lane 2, crude protein extract from *S. cerevisiae* MGY70. Lane 3, crude protein extract from *S. cerevisiae* MGY70-myr induced with galactose. Lane 4, fractions eluted from EZCatch^Tm^ GST-Spin column. (**D**) Western blot, using anti-GST antibody. Lane 1, molecular mass marker (Precision Plus Protein Dual Xtra Standards). Lane 2, crude protein extract from *S.* *cerevisiae* MGY70. Lane 3, crude protein extract from *S. cerevisiae* MGY70 induced with galactose. Lane 4, fractions eluted from EZCatch^Tm^ GST-Spin column. Western Blot images correspond to autoradiographs overlapped to the nitrocellulose membrane. The numbers on the left correspond to molecular mass in kDa.

Electrophoretic analysis of total proteins from an IPTG induced culture of *E. coli* BL21(DE3)-myr, revealed overproduction of a ~48 kDa polypeptide, which was not observed in the non-transformed strain, nor in the non-induced transformed strain (Figure 1A, lanes 2, 3, 4). This polypeptide band corresponds to the monomer of broccoli myrosinase, a product of the expression of the cDNA contained in the vector pET22-b(+)-myr, which was later confirmed with the purification of said protein by chromatography on NTA-Ni columns (Figure 1A, lanes 5, 6, and 7), and analysis by Western Blot (Figure 1B). Regarding the myrosinase purification process, the percentage yield obtained was 90% in the second and third elution at an imidazole concentration of 1 M, from the NTA-Ni column (Table 1). These data show that the loss of total enzymatic activity (U) is not significant, and the specific activity increases 5 times from the crude extract until the purification of the protein of interest.

In galactose-induced *S. cerevisiae* MGY70-myr, production of a polypeptide of ~83 kDa was observed, which was not observed in the non-transformed strain (Figure 1C, lanes 2 and 3). However, this polypeptide band does not correspond to the broccoli myrosinase monomer, since the purification of said protein preparation using an EZCatchTM GST-Spin column revealed a ~70 kDa polypeptide that comigrates with other proteins present in the non-induced strain (Figure 1C, lane 4). The nature of this polypeptide band was subsequently confirmed by Western Blot using anti-GST antibodies (Figure 1D). In the myrosinase purification process, a yield of 68% was obtained in the fraction collected from the Glutathione Sepharose column (Table 1). The data shows that the specific activity increases 4-fold from the crude extract until the purification of the protein of interest.

### 3.2. Effect of Temperature and pH on Myrosinase Activity

**Figure 2:**
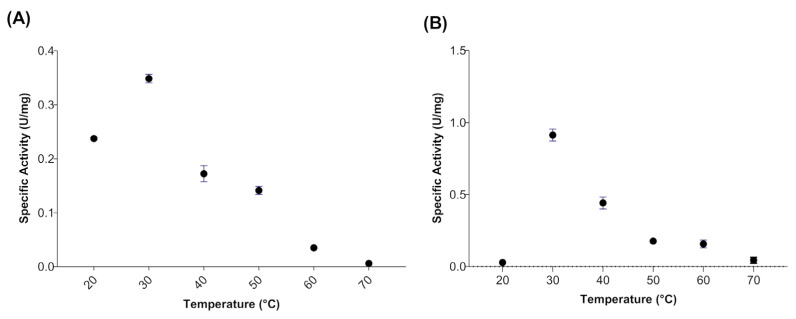
Temperature effect on activity of myrosinase from *E. coli* BL21(DE3)-myr (**A**) and *S. cerevisiae* MGY70-myr (**B**). The activity was determined in the range 20–70 °C at pH 7.0. Reaction mixture contained 0.05 mg protein and 0.1 mM sinigrin.

**Figure 3:**
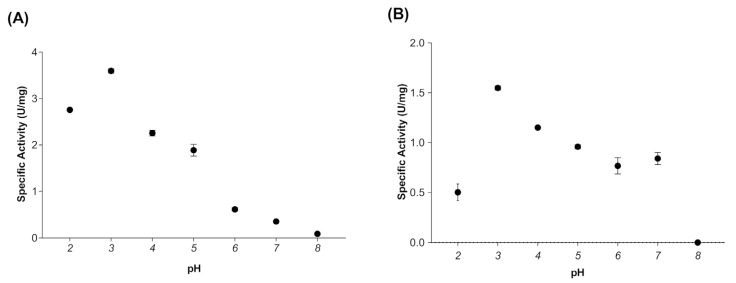
Effect of pH on activity of myrosinase from *E. coli* BL21(DE3)-myr (**A**) and *S. cerevisiae* MGY70-myr (**B**). The activity was determined in the pH range 2–8 at 30 °C. Reaction mixture contained 0.05 mg protein and 0.1 mM sinigrin.

When evaluating the effect of temperature on the activity of myrosinase produced in *E. coli* BL21(DE3)-myr, the results revealed that the enzyme remains active in a range from 20 to 50 °C, obtaining maximum activity at 30 °C (Figure 2A), while at 60 and 70 °C a drastic loss of activity was observed. On the other hand, the myrosinase produced in *S. cerevisiae* MGY70-myr, maintained its activity in a range of 30 to 60 °C, while at 70 °C it completely lost its activity (Figure 2B). Both myrosinases showed activity in the pH range from 2 to 7, obtaining the maximum activity at pH 3 (Figure 3A,B).

### 3.3. Kinetic Behavior of Recombinant Myrosinase

**Figure 4:**
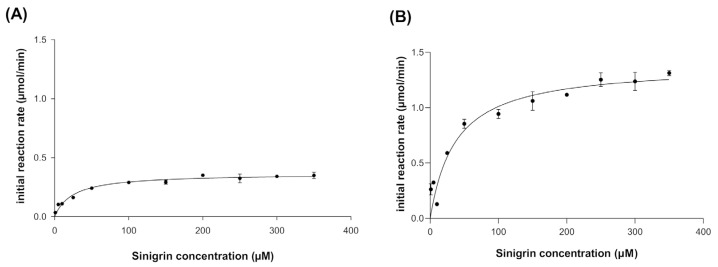
Kinetic behavior of myrosinase produced in *E. coli* BL21(DE3)-myr (**A**) and *S. cerevisiae* MGY70-myr (**B**). Assays were conducted at 30 °C in 33 mM pH 7.0 phosphate buffer. The reaction mixture contained 0.05 mg protein.

The kinetic behavior of broccoli myrosinase produced in *E. coli* BL21(DE3)-myr and *S. cerevisiae* MGY70-myr was evaluated at pH 7.0 and 30 °C, since these are the optimal conditions for the production of bioactive metabolites. The experimental data were fitted to the Michaelis–Menten model, where it was observed that at high concentrations of the substrate sinigrin, the reaction rate asymptotically approaches the maximum rate (Figure 4A,B). The most relevant adjustment parameters considered are K_m_, V_max_, *k*_cat_ and *k*_cat_/K_m_, with values of 24.32 ± 3.47(μM), 0.364 ± 0.011 (μmol/min), 7.288 ± 0.2152 (s^−^^1^), and 0.3 (s^−^^1^ μM^−^^1^), respectively, for myrosinase produced in *E. coli* BL21(DE3)-myr, and values of 37.37 ± 7.51 (μM), 1.388 ± 0.06 (μmol/min), 27.77 ± 1.323 (s^−^^1^) and 0.743 (s^−^^1^ μM^−^^1^), respectively, for myrosinase produced in *S. cerevisiae* MGY70-myr (Table 2).

### 3.4. Determination of the Molecular Mass of Native Myrosinases

**Figure 5:**
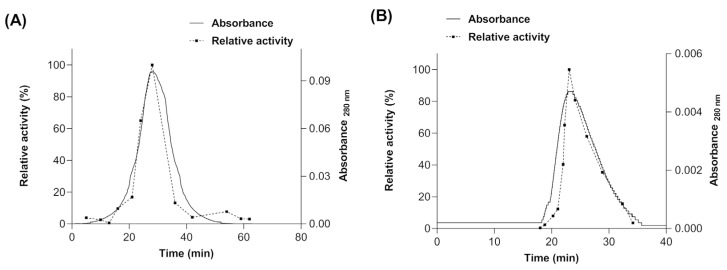
Size Exclusion Chromatography of purified fractions obtained from NTA-Ni y EZCatch^Tm^ GST-Spin columns. (**A**) Myrosinase produced in *E. coli* BL21(DE3)-myr. (**B**) Myrosinase produced in *S. cerevisiae* MGY70-myr.

The molecular mass of the native myrosinase produced in *E. coli* BL21(DE3)-myr and *S. cerevisiae* MGY70-myr was determined by molecular size exclusion chromatography (SEC). For myrosinase produced in *E. coli* BL21(DE3)-myr, a single absorbance peak was observed at 28 min (Figure 5A), whose fraction had a relative activity of 100% using sinigrin as substrate, and according to the calibration curve (data not shown) would correspond to a protein of molecular mass ~49 kDa. Therefore, these results indicate that there is no quaternary structure formation. For the myrosinase produced in *S. cerevisiae* MGY70-myr, a single absorbance peak was observed at 23 min (Figure 5B), whose fraction had a relative activity of 100% using sinigrin as substrate, and according to the calibration curve it would correspond to a protein with a molecular mass of ~73 kDa. The results preliminarily suggest that there is no quaternary structure formation.

### 3.5. Proteolytic Digestion and Deglycosylation of Myrosinase Produced in S. cerevisiae MGY70-myr

**Figure 6:**
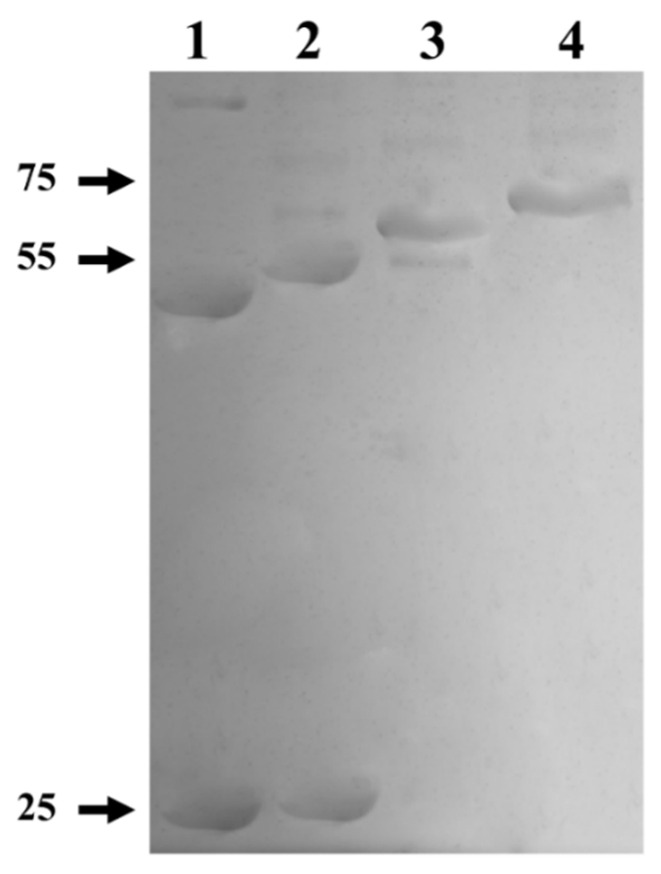
SDS-PAGE from the digestion and deglycosylation products of myrosinase produced in S. cerevisiae MGY70-myr. Lane 1: Purified protein fraction from the anti-GST column, deglycosylation with Endo H, and digestion with thrombin protease. Lane 2: Purified protein fraction from the anti-GST column and digestion with thrombin protease. Lane 3: Purified protein fraction from the anti-GST column and deglycosylation with Endo H. Lane 4: Purified protein fraction from the anti-GST column. Reaction mixtures contained 0.05 mg protein. The numbers on the left correspond to molecular mass in kDa.

To elucidate whether the formation of the quaternary structure is prevented by a misfolding of the protein, due to the fact that it has a GST-tag at the amino end, a proteolytic treatment with thrombin protease was performed to remove the GST-tag. On the other hand, a treatment with Endoglycosidase H (deglycosylation) was carried out in order to determine if the glycosylations present in myrosinase affect its enzymatic activity. The results after each treatment revealed changes in the electrophoretic mobility of the enzyme (Figure 6). In lane 1, a band of ~48 kDa was observed corresponding to the deglycosylated myrosinase enzyme without GST-tag, which when removed migrates as a polypeptide of ~26 kDa. In lane 2, a band of ~50 kDa was observed, that corresponds to the enzyme previously digested with thrombin protease. In lane 3, a band of ~68 kDa was observed, which corresponds to the enzyme deglycosylated previously with Endo H. Finally, in lane 4, a band of ~70 kDa corresponding to myrosinase purified from a Glutathione Sepharose column was observed.

**Figure 7:**
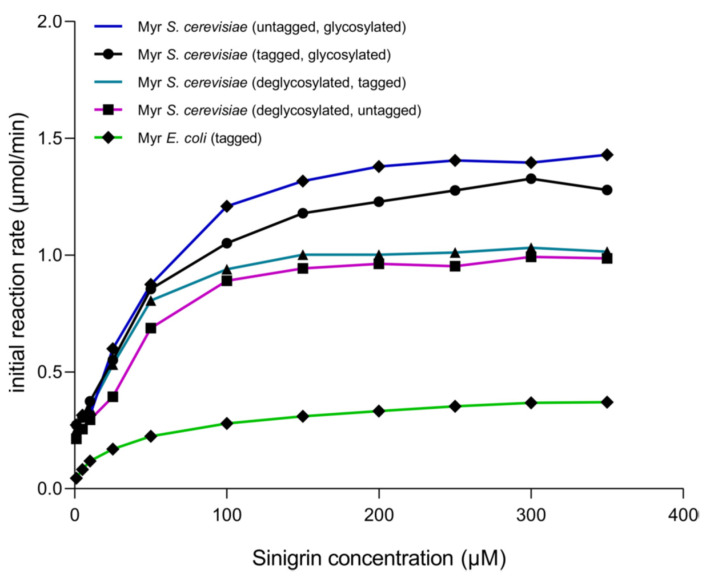
Initial reaction rate of myrosinase produced in *S. cerevisiae MGY70-myr* subjected to different treatments, in comparison with myrosinase produced in *E. coli* BL21(DE3)-myr. Assays were conducted at 30 °C in sodium phosphate buffer pH 7.0. The reaction mixtures contained 0.05 mg protein.

After each stage of proteolytic digestion and deglycosylation, it was determined how these treatments affected enzyme activity. The kinetic behavior shows that the enzyme produced in *S. cerevisiae* MGY70-myr adjusts to Michaelis–Menten model in all treatments (Figure 7). Also, *k*_cat_/V_max_ is higher when the enzyme is deglycosylated and with GST-tag, as compared with the untreated enzyme (Table 2), however, the difference is not significant. When comparing these treatments with the results obtained previously with myrosinase produced in *E. coli* BL21(DE3)-myr, it is observed that the V_max_ is up to 4 fold higher in myrosinase produced in *S. cerevisiae* MGY70-myr depending on the treatment. On the other hand, there is no significant difference in the turnover number in each treatment, however, in myrosinase produced in *E. coli* BL21(DE3)-myr the *k*_cat_ is up to 4fold lower in relation to myrosinase produced in *S. cerevisiae* MGY70-myr depending on the treatment (Table 2).

**Figure 8:**
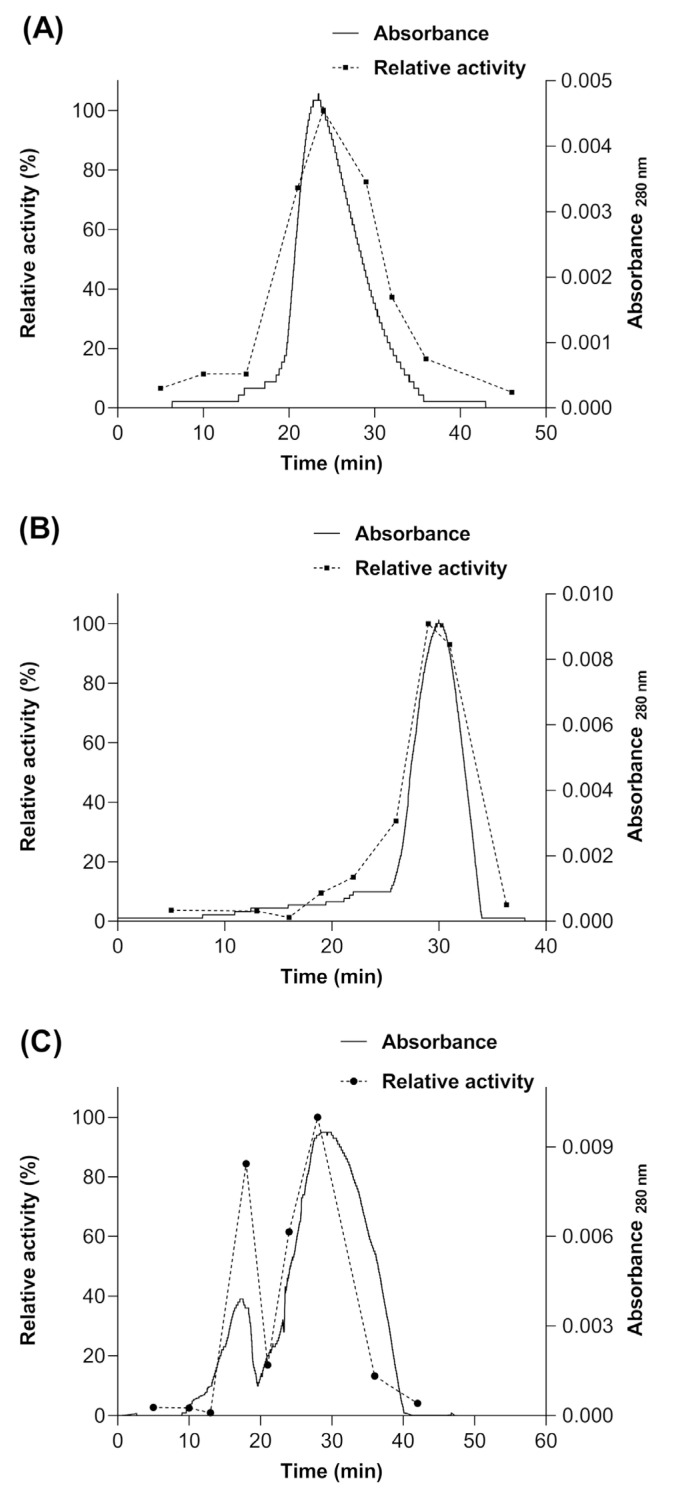
Size Exclusion Chromatography of myrosinase produced in *S. cerevisiae* MGY70-myr subjected to different treatments. (**A**) deglycosylation with Endo H; (**B**) deglycosylation with Endo H and digestion with thrombin protease, (**C**) digestion with thrombin protease.

Subsequently, a size exclusion chromatography (SEC) was performed after each treatment mentioned above, in order to determine if the enzyme produced in *S. cerevisiae* MGY70-myr acquires some type of quaternary structure or if it remains in its monomeric conformation. The results show that a portion of the population of molecules of the myrosinase enzyme produced in *S. cerevisiae* MGY70-myr acquires a quaternary structure when the GST-tag is removed by proteolytic digestion (Figure 8C). However, to acquire this type of structuring, the enzyme must be glycosylated (Figure 8C), since being deglycosylated and not having the GST-tag, 100% of the population of myrosinase molecules remains in its monomeric conformation (Figure 8B). The molecular mass of the proteins present in the fractions of the main chromatographic peak was calculated through a calibration curve with protein standards. The results obtained were: for treatment with Endoglycosidase H ~72 kDa (Figure 8A), treatment with Endoglycosidase H and thrombin protease ~49 kDa (Figure 8B) and treatment with thrombin protease ~106 kDa (first peak) ~53.1 kDa (second peak) (Figure 8C).

Finally, the specific activity of myrosinase was determined, expressed as a percentage for each fraction collected from the Sephacryl S-200 column and in each treatment mentioned above. In the treatment with thrombin protease, it was observed that the maximum activity is present in the myrosinase monomer and in 84% for the dimer (Figure 8C). For the other treatments, only a peak was observed in the chromatogram, coinciding with the maximum activity of the enzyme, and that according to their elution times, they would correspond to the monomeric form of the enzyme and to myrosinase without treatment (Figure 5A,B).

## 4. Discussion

With the purification process of myrosinase produced in *E. coli* BL21(DE3)-myr and *S. cerevisiae* MGY70-myr, an enzyme preparation was obtained with a specific activity close to 6.4 and 5.2 (U/mg), respectively, unlike that obtained by other authors for myrosinases from other origins, such as myrosinase purified from *Lepitidium latifolium* leaves, with a specific activity of 3.12 (U/mg) [16] and myrosinase from a different origin than vegetable, obtained from the *Brevicoryne brassicae* aphid, with a specific activity of 0.9 (U/mg) [19]. These results were shown to be significantly lower than those obtained in this work for both enzymes, and this is because the purification processes are carried out by means of protein precipitation with ammonium sulfate, gel filtration and affinity chromatography with concanavalin A resins. Our results demonstrated that protein purification by affinity chromatography, with specific resins for His-tag or GST-tag, it is much more efficient and specific than the methods mentioned above. Additionally, it should be considered that our expression strategies allow the enzyme to be overproduced. This has been observed in most heterologous expression systems and therefore they are used in order to obtain a correlation between the purity of an enzyme (U/mg) and the amount (mg) of pure enzyme, either for analysis by X-ray diffraction (≈10 U/mg) [30], subsequent studies in site-directed mutagenesis (0.05–0.075 U/mg) [31], studies with DNA binding proteins or molecular genetics (0.97–1 U/mg) [32]. Other authors have described the use of heterologous expression systems for the production of recombinant enzymes such as β- glucosidases [37], and sterol esterase/lipase obtained from different hosts such as *Pichia pastoris* and *S. cerevisiae* [38].

The study of the structural organization of myrosinase produced in *S. cerevisiae* MGY70-myr revealed that a portion of the population of protein molecules acquires a homodimer quaternary structure when the GST-tag was removed, maintaining the glycosylations produced posttranslationally in the yeast. Because the GST-tag constitutes a polypeptide moiety of appreciable size, it is possible that its presence in the myrosinase monomer is masking important regions of interaction between subunits for homodimer formation. Similar results were not observed in myrosinase produced in *E. coli* BL21(DE3)-myr, since the enzyme is not glycosylated, and several studies have described the importance of glycosylations in the formation of quaternary structure, due to hydrogen bonds interactions between the monomeric subunits [39,40]. Our results differ from those described so far with those obtained with broccoli myrosinase purified from vegetable, since it has been reported that in this case the native enzyme is a homotrimer of ~157 kDa [24]. Other myrosinases also possess a homodimeric quaternary structure [41,42,43] and it has also been described that in some cases high molecular weight protein complexes are formed [12]. The myrosinase produced in *E. coli* BL21(DE3)-myr maintains its activity in a temperature range between 20 to 50 °C, while it loses approximately 85% of its activity at 60 °C and completely at 70 °C. Naturally, at these last temperatures, reasonably high for a macromolecule of a mesophyllic organism, the interactions that stabilize the three-dimensional structure of the enzyme are broken until it is completely denatured. On the other hand, the enzyme produced in *S. cerevisiae* MGY70-myr maintains at least 25% of its activity at 60 °C and it has been described that the native broccoli enzyme maintains activity in a wider range of temperatures (20 to 70 °C) [24]. The differences in thermal stability of myrosinase purified from broccoli and that produced in *S. cerevisiae* MGY70-myr compared to that produced in *E. coli* BL21(DE3)-myr, could be due to the presence of glycosylations in the first two, which would give them greater structural stability and therefore, higher resistance to denaturation. Probably from a chemical point of view, glycosylated enzymes have a greater amount of intramolecular hydrogen bonding interactions and to break them, a greater amount of energy is needed.

The myrosinases produced in *E. coli* BL21(DE3)-myr and *S. cerevisiae* MGY70-myr maintain their activity in a pH range of 2 to 7, reaching their maximum activity at pH 3. The same occurs with native broccoli myrosinase [24], however, most of the myrosinases described present their maximum enzymatic activity at neutral pH [1]. This implies differences in the catalytic mechanism of the enzyme, probably due to the presence of a glutamate residue (Glu 429) in broccoli myrosinase, which was elucidated by molecular docking as one of those responsible for the cleavage of the β-thioglycosidic bond [25]. This residue could act through an acid/base mechanism in the hydrolysis of the β-thioglucosidic bond, unlike what occurs in the myrosinase of *S. alba* [20], where a water molecule nucleophilically attacks the anomeric carbon of the glucose molecule with the unpaired electrons of its oxygen atom, thus allowing the hydrolysis of the intermediary glycosyl-enzyme.

Some interesting aspects were observed in the kinetic analysis of the myrosinases produced in *E. coli* BL21(DE3)-myr and *S. cerevisiae* MGY70-myr. The myrosinase produced in *S. cerevisiae* MGY70-myr showed differences, although not significant, in the K_m_ values when it was treated with Endoglycosidase H and thrombin protease.

The myrosinase produced in *S. cerevisiae* MGY70-myr presented values up to 4 times higher in *k*_cat_ and *k*_cat_/K_m_ compared to the enzyme produced in *E. coli*. According to these parameters, we can conclude that the enzyme produced in *S. cerevisiae* MGY70-myr is a better biocatalyst than the myrosinase produced in *E. coli* BL21(DE3)-myr, since it has a greater catalytic potential associated with the turnover number and its catalytic efficiency. Similar kinetic parameters such as K_m_, V_max_ and *k*_cat_ have been described in other recombinant myrosinases produced in *A. thaliana* [15,44] and *C. papaya* [32]. In other myrosinases obtained from plants, a lower catalytic efficiency has been observed, as in the case of the enzymes of *A. rusticana* and *Lepidium latifolium* L [13,16].

Regarding the kinetic model, both recombinant enzymes fitted to the Michaelis–Menten model. However, the native broccoli myrosinase fit a kinetic model of substrate inhibition [24,25]. Nevertheless, the results of this work are in agreement with those published for most of the myrosinases described, such as those of *B. thuringiensis* [18] *Brassica oleracea* var. capitata L. [45], *C. papaya* [32] and *B. napus* [46].

## 5. Conclusions

With the expression in *E. coli* and *S. cerevisiae* of the broccoli myrosinase cDNA, it was possible to obtain the recombinant enzyme from both microorganisms, determine some optimal physical and chemical parameters such as temperature and pH, elucidate its kinetic behavior and obtain information on the quaternary structure and the importance of glycosylations in stabilizing the homodimeric structure of the enzyme obtained from *S. cerevisiae*, in addition to comparing the catalytic efficiency of both enzymes, being approximately 4 times higher that of the myrosinase obtained from *S. cerevisiae*. The results are promising and taking advantage of this knowledge, it will be possible to design myrosinase enzyme overproduction strategies, from the recombinant clone of *S. cerevisiae*, then immobilize the enzyme in an inert matrix and thus continuously produce sulforaphane, a metabolite with important medicinal properties.

## Figures and Tables

**Table 1:** Purification steps of myrosinases from *E. coli* BL21(DE3)-myr and *S. cerevisiae* MGY70-myr.

Stage	Total Protein Concentration (mg/mL)	Total Activity * (U)	Specific Activity (U/mg)	Accumulated Yield (%)
**Myrosinase produced in *E. coli***				
Crude extract	3.526	5.121	1.452	100
Total proteins eluted with wash buffer	0.963	4.922	5.112	97.5
Elution of proteins retained in the column	0.721	4.619	6.407	90.2
**Myrosinase produced in *S. cerevisiae***				
Crude extract	5.271	5.639	1.070	100
Total proteins eluted with wash buffer	3.210	3.848	1.199	68.2
Elution of proteins retained in the column	0.512	2.706	5.287	48.0

* Enzyme activity was determined as 0.1 mM sinigrin hydrolysis rate.

**Table 2:** Kinetic parameters estimated for myrosinases produced in *S. cerevisiae* MGY70-myr and *E. coli* BL21(DE3)-myr.

Myrosinase From:	K_m_ (μM)	V_max_ (μmol/min)	*k*_cat_ (s^−1^)	*k*_cat_/K_m_ (s^−1^ μM^−1^)
*S. cerevisiae* (glycosylated, with GST tag)	37.37 ± 7.51	1.388 ± 0.066	27.77 ± 1.323	0.743
*S. cerevisiae* (glycosylated, without GST tag)	25.92 ± 4.563	1.079 ± 0.047	21.58 ± 0.801	0.833
*S. cerevisiae* (deglycosylated, with GST tag)	17.44 ± 2.828	1.106 ± 0.034	22.11 ± 0.6796	1.268
*S. cerevisiae* (deglycosylated, without GST tag)	35.82 ± 5.767	1.595 ± 0.060	31.9 ± 1.2	0. 891
*E. coli*	24.32 ± 3.47	0.364 ± 0.011	7.288 ± 0.2152	0.300

## Data Availability

Data are contained within the article.

## Supplementary Materials

The following supporting information can be downloaded at: https://www.mdpi.com/article/10.3390/biom12020233/s1, Supplementary file, Figure S1. Enzymatic activity of myrosinase produced in *S. cerevisiae* MGY70-myr after different treatments, in comparison with myrosinase produced in *E. coli* BLD21(DE3)-myr. The kinetic constants K_m_ and V_max_ were calculated from the Lineweaver-Burk plot, fitting a linear regression for each reciprocal. The assays were performed at 30 °C, in sodium phosphate buffer pH 7. The reaction mixture contained 0.05 mg of protein.
