# Supplementary material for: Broccoli Myrosinase cDNA Expression in Escherichia coli and Saccharomyces cerevisiae
Source: Biomolecules. 2022 Jan 30;12(2):233. doi: 10.3390/biom12020233 (PMC8961631; doi:10.3390/biom12020233)
Supplement: Supplementary file 1 [file biomolecules-12-00233-s001.zip › biomolecules-1570445-supplementary.pdf]

biomolecules

MDPI

Supplementary material

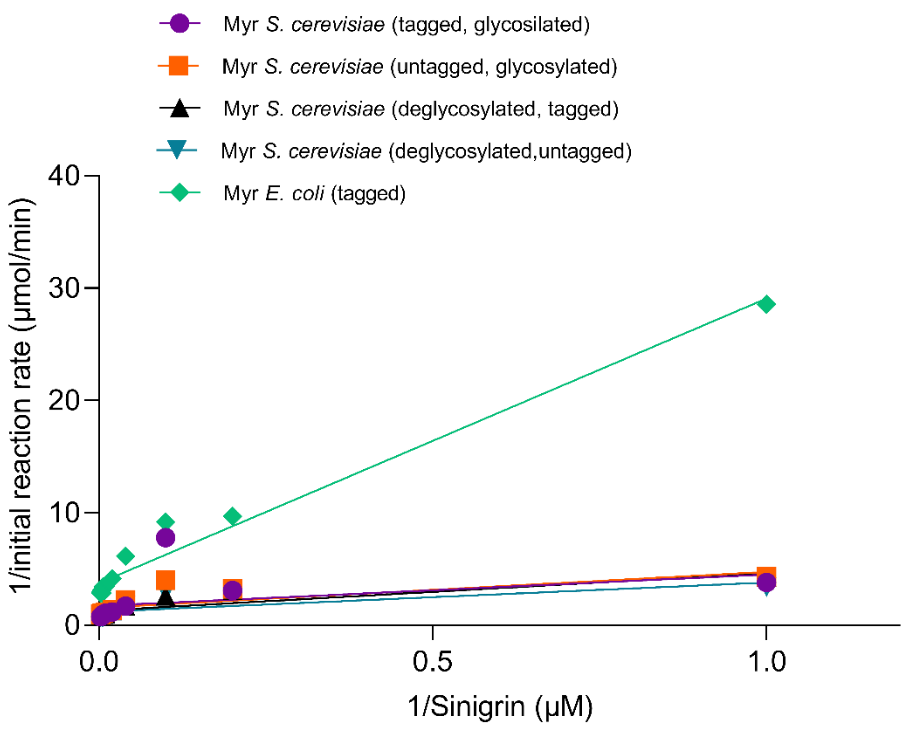

Figure S1. Enzymatic activity of myrosinase produced in *S. cerevisiae* MGY70-myr after different treatments, in comparison with myrosinase produced in *E. coli* BLD21(DE3)-myr. The kinetic constants K_m and V_{max} were calculated from the Lineweaver-Burk plot, fitting a linear regression for each reciprocal. The assays were performed at 30 °C, in sodium phosphate buffer pH 7. The reaction mixture contained 0.05 mg of protein.
